# Functional studies of intimin *in vivo* and *ex vivo*: implications for host specificity and tissue tropism

**DOI:** 10.1099/mic.0.2006/003467-0

**Published:** 2007-04

**Authors:** Rosanna Mundy, Stephanie Schüller, Francis Girard, John M. Fairbrother, Alan D. Phillips, Gad Frankel

**Affiliations:** 1 Division of Cell and Molecular Biology, Imperial College London, London, UK; 2 Centre for Paediatric Gastroenterology, Royal Free and University College Medical School, London, UK; 3 Groupe de Recherche sur les Maladies Infectieuses du Porc (GREMIP), Faculté de Medicine Vétérinaire, Université de Montréal, St-Hyacinthe, Canada

## Abstract

Intimin is an outer-membrane adhesin that is essential for colonization of the host gastrointestinal tract by attaching and effacing pathogens including enteropathogenic *Escherichia coli* (EPEC), enterohaemorrhagic *E. coli* (EHEC) and *Citrobacter rodentium* (CR). The N-terminus of intimin from the different strains is highly conserved while the C-terminus, which harnesses the active receptor-binding site, shows sequence and antigenic polymorphism. This diversity was used to define a number of distinct intimin types, the most common of which are *α*, *β* and *γ*. Intimin binds the type III secretion system effector protein Tir. However, a large body of evidence suggests that intimin also binds a host-cell-encoded receptor(s) (Hir), and interaction of different intimin types with Hir contributes to tissue and host specificity. The aims of this study were to compare the activity of the major intimin types (*α*, *β* and *γ*) *in vivo* and *ex vivo*, using the CR mouse model and *in vitro* organ culture (IVOC), and to determine their exchangeability. The results confirm that intimin *γ* is not functional in the CR mouse model. In the pig, intimin *β* can substitute for EPEC intimin *α* but when placed in an EHEC O157 : H7 background it does not produce an intimin *α*-like tropism, although some adhesion to the small and large intestine was observed. In contrast, in human IVOC, intimin *β* in an EHEC background produces small intestinal colonization in a similar manner to intimin *α*.

## INTRODUCTION

Enteropathogenic *Escherichia coli* (EPEC) and enterohaemorrhagic *E. coli* (EHEC) are important causes of acute gastroenteritis in humans (Nataro & Kaper, 1998). EPEC is a frequent cause of infantile diarrhoea in the developing world (Chen & Frankel, 2005) while EHEC causes a wide spectrum of illnesses ranging from mild diarrhoea to severe diseases, such as haemorrhagic colitis and haemolytic uraemic syndrome (HUS). HUS is the leading cause of acute renal failure in children, and is associated with the production of potent Shiga toxins (Stx) (Thorpe, 2004). Strains of EHEC belonging to serogroup O157 are most commonly associated with severe human disease (Mead *et al.*, 1999). However, infections with other EHEC strains, such as those of serogroups O26 and O103, are on the rise (Brooks *et al.*, 2005).

EHEC and EPEC exhibit narrow host specificity and, given that mice are by and large resistant to symptomatic infection, a difficulty with studying EPEC and EHEC pathogenesis is the lack of a simple small animal model to simulate an *in vivo* situation. For this reason, infection of mice with *Citrobacter rodentium* (CR), a natural mouse pathogen that shares many of its virulence factors and mechanism of colonization with EPEC and EHEC, has become a popular surrogate model for *in vivo* studies (Mundy *et al.*, 2005).

When adhering to intestinal epithelial cells EPEC, EHEC and CR subvert cytoskeletal processes to produce a histopathological feature known as an attaching and effacing (A/E) lesion (Nataro & Kaper, 1998; Garmendia *et al.*, 2005; Mundy *et al.*, 2005). This is characterized by localized destruction of brush border microvilli and intimate attachment of the bacteria to the plasma membrane of the host epithelial cells. The capacity to form A/E lesions is encoded mainly on a pathogenicity island termed the locus of enterocyte effacement (LEE) (McDaniel *et al.*, 1995), which encodes components of a type III secretion system (Jarvis *et al.*, 1995), chaperones, translocator and effector proteins (Garmendia *et al.*, 2005) as well as the outer-membrane adhesin intimin (Jerse *et al.*, 1990).

Intimin, the product of the LEE *eae* gene, was the first to be associated with A/E lesion formation (Jerse *et al.*, 1990). Analysis of intimin sequences from different EPEC and EHEC strains revealed that while the first ∼700 amino acids are highly conserved (over 97 % identity), the C-terminal 280 amino acids (Int280) are variable; the active receptor-binding site of intimin resides within the Int280 domain (Frankel *et al.*, 1994). Indeed, when expressed as an N-terminal fusion with carrier proteins, Int280 binds directly to epithelial cells (Frankel *et al.*, 1994) and interacts with nucleolin (Sinclair & O'Brien, 2004) and integrin (Frankel *et al.*, 1996a). Int280 also binds the LEE-encoded effector protein Tir, which connects the extracellular bacterium to the host cell cytoskeleton (Kenny *et al.*, 1997).

The solution (Kelly *et al.*, 1999) and crystal (Luo *et al.*, 2000) 3D structure of Int280*α* revealed that the polypeptide comprises a series of three globular modules with a distinct organization. The two domains (D1–2) closest to the bacterial cell surface comprise *β*-sheet sandwiches and structurally resemble immunoglobulin (Ig)-like folds. A third domain (D3) located at the C-terminal tip of the molecule is formed by the 76 amino acid disulfide loop that shows some structural similarity to C-type lectin. The Cys residues (C860 and C937) forming the disulfide loop are totally conserved among the different intimin types and are required for cell-binding activity (Frankel *et al.*, 1995) and A/E lesion formation (Frankel *et al.*, 1998).

Based on polymorphism within Int280, we reported the existence of several different classes of intimin, the most common of which are *α*, *β* and *γ* (Adu-Bobie *et al.*, 1998). In particular, intimin *α* is associated with the distinct evolutionary lineage of EPEC known as EPEC-1, intimin *γ* is associated with EHEC O157 : H7, while intimin *β* appears to be the most ubiquitous type and is found among human and animal pathogens including EPEC-2, EHEC-2 and CR. A large body of evidence suggests that the different intimin types influence host specificity and tissue tropism (Girard *et al.*, 2005; Phillips & Frankel, 2000; Tzipori *et al.*, 1995). *In vivo* experiments using gnotobiotic piglets revealed that EHEC O157 : H7, which expresses intimin *γ*, is associated with extensive colonization and destruction of the large intestinal epithelium while EPEC O127 : H6, which expresses intimin *α*, colonizes both the small and large intestine (Tzipori *et al.*, 1995). Importantly, complementation of an *eae* mutant of EHEC O157 : H7 with *eae_α_* alters the pattern of colonization so that colonization was seen in the small and large intestine in a similar manner to EPEC (Tzipori *et al.*, 1995). Using human and porcine intestinal *in vitro* organ culture (IVOC) we have shown that as in the gnotobiotic piglet model, intimin exchanges in both EHEC O157 : H7 and EPEC O127 : H6 resulted in alteration in tissue tropism (Fitzhenry *et al.*, 2002a; Girard *et al.*, 2005; Phillips & Frankel, 2000). In this study we compared the functionality of the different intimin types *in vivo* and *ex vivo* and performed further intimin exchange studies evaluating the function of CR intimin *β* in EPEC and EHEC isolates.

## METHODS

### Bacterial strains and plasmids.

The bacterial strains used in this study were wild-type CR strain ICC169, EPEC O127 : H6 (strain E2348/69), EHEC O157 : H7 strain 85-170 and their *eae* deletion mutants, strains DBS255 (Schauer & Falkow, 1993), CVD206 (Donnenberg & Kaper, 1991) and ICC170 (Fitzhenry *et al.*, 2002a). The plasmids used in this study are listed in Table 1. Plasmid pCVD438 is a pACYC184 vector harbouring the intimin *α* gene from E2348/69 (Donnenberg & Kaper, 1991). pICC55 is a derivative of pCVD438 in which the 3′ end of the *eae* gene, encoding the Int280*α* domains, was substituted with a fragment of *eae_γ_* from EHEC encoding Int280*γ* (Fitzhenry *et al.*, 2002a). Bacteria were grown at 37 °C in either high-glucose Dulbecco's Modified Eagle's Medium (DMEM) or LB and where appropriate, nalidixic acid, kanamycin and chloramphenicol were added to final concentrations of 100, 50 and 35 μg ml^−1^ respectively.

### Replacing the Int280*α* coding region in pCVD438 with CR Int280*β*.

**Fig. 1. f1:**
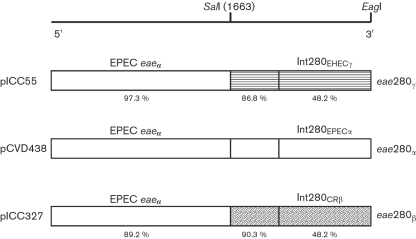
Schematic representation (not to scale) showing the construction of recombinant intimin *β* and *γ*. The C-terminal regions including the receptor-binding regions of intimin *α* (*eae*280*_α_*), intimin *β* (*eae*280*_β_*) and intimin *γ* (*eae*280*_γ_*) are represented by different shading. The percentage amino acid identity of each region of intimin *β* and intimin *γ* compared to that of intimin *α* is indicated. The position of the restriction site, *Sal*I, used to construct the recombinant intimin is given in nucleotides and the position corresponding to *Sal*I in intimin is represented as a solid line.

A schematic representation of the strategy used to replace the Int280*α* region of pCVD438 with Int280*β* from CR is shown in Fig. 1. Two unique restriction endonuclease sites located in pCVD438 were used, a conserved *Sal*I site located upstream of the Int280 domain (position 1663 of the *eae* gene) and an *Eag*I site located downstream of the TAA stop codon and within the pACYC184 vector plasmid (Frankel *et al.*, 1998). The DNA fragment between the *Sal*I site and the 3′ end of the *eae* gene encoding intimin *β* from CR strain ICC169 was amplified by PCR using a forward primer (CReaefor2 5′-CCGTTCTGTCGAATGGTCAAGTAG-3′) and a CR *eae_β_*-derived reverse primer overlapping the end of the gene and including an *Eag*I restriction site (CReaerev1EagI 5′-CGGCCGTACACAGAATTATGGACAGTCCCG-3′). The amplified *eae* fragment, flanked by *Sal*I and *Eag*I restriction sites, was used to replace the corresponding fragments of pCVD438 as previously described (Frankel *et al.*, 1998) (Fig. 1). Following confirmation by DNA sequencing, the modified plasmid, pICC327, was transformed into the *eae* deletion mutants of EPEC, EHEC and CR, strains CVD206, 85-170 and DBS255, respectively, by electroporation.

### Infection of mice.

Male, specific-pathogen-free C3H/Hej mice that were 6–8 weeks old were purchased from Harlan Olac (Bicester, UK). All the mice were housed in individual ventilated cages with free access to food and water. Bacteria were grown to stationary phase in LB broth containing the appropriate antibiotic. A 1 ml sample of the broth was centrifuged and the bacterial pellet was resuspended in 2.5 ml PBS. Mice were orally inoculated with 200 μl of the bacterial suspension (approx. 1×10^8^ c.f.u. per mouse) by gavage. The viable count of the inoculum was determined by retrospective plating on LB agar containing appropriate antibiotics. Each bacterial strain was tested in independent experiments at least twice using groups of at least five mice per strain. Stool samples were recovered aseptically at various times after inoculation and the number of viable bacteria (c.f.u.) per g stool was determined by plating samples onto LB agar containing appropriate antibiotics. Mice were euthanased by cervical dislocation 8 days post-challenge. Colon and caecum were removed from each mouse, photographed and weighed after removal of stools. The colons were then homogenized mechanically with a Seward 80 stomacher (Seward, London, UK) and the numbers of viable bacteria per colon were determined by plating onto LB agar containing the appropriate antibiotics.

### Statistical analysis.

All results are presented as the group mean±sem. One-way analysis of variance (ANOVA) was performed to test any differences between strains. Analysis was performed using Minitab Statistical Software, release 10.5 Xtra.

### Immunofluorescence staining of frozen tissue.

Frozen distal mouse colons were embedded in OCT compound (Sakura) and serial sections of 8 μm were cut with an MTE cryostat (SLEE Technik). Sections were picked up on poly-l-lysine-coated slides and air-dried. After formalin fixation for 10 min, tissue sections were blocked with 0.5 % BSA and 2 % normal goat serum in PBS for 20 min. Slides were incubated in primary antibody (rabbit anti-Tir 1 : 200 or rabbit anti-CR 1 : 1000) for 60 min at room temperature, washed and incubated in Alexa Fluor 488-conjugated goat anti-rabbit IgG (Molecular Probes) for 30 min. Actin filaments were stained with Alexa Fluor 647 phalloidin (Molecular Probes). Counterstaining of bacteria and cell nuclei was performed using propidium iodide (Sigma). Sections were analysed with a Radiance 2100 confocal laser scanning microscope equipped with an argon-krypton laser and a red diode (Bio-Rad).

### Human *in vitro* organ culture (IVOC).

Tissue was obtained with fully informed parental consent and local ethical committee approval using grasp forceps during routine endoscopic (Fujinon EG/EC-41 paediatric endoscope) investigation of gastrointestinal complaints. Proximal small intestinal mucosal biopsies (patients' age 72, 103, 132 and 181 months) from the fourth part of the duodenum which appeared macroscopically normal were taken for organ culture experiments. Light microscopy subsequently showed no histological abnormality. IVOC infections were performed as described previously (Hicks *et al.*, 1998). In each experiment an un-inoculated sample (to exclude endogenous bacterial adhesion) and a positive control were included. Samples were fixed with 2.5 % glutaraldehyde, post-fixed in 1 % aqueous osmium tetroxide and processed for viewing by a JEOL JSM 5300 scanning electron microscope (SEM).

### Collection and culture of porcine intestinal IVOC explants.

Piglets were cared for in accordance with the Guidelines of the Canadian Council for Animal Care. The porcine intestinal IVOC model was used as previously described (Girard *et al.*, 2005). Briefly, segments of the duodenum, jejunum, ileum, caecum and colon were obtained from colostrum-deprived newborn piglets of a conventional herd. Piglets were tranquillized before being euthanased as described elsewhere (Girard *et al.*, 2005). Explants were inoculated three times at hourly intervals with 50 μl broth culture applied to the mucosal surface, and incubated at 37 °C on a rocker in a 95 % O_2_/5 % CO_2_ atmosphere for 8 h. Sample explants were processed for SEM as previously described (Girard *et al.*, 2005).

### Histopathology.

After culture, porcine explants were rinsed thoroughly in sterile PBS and fixed in 10 % buffered formalin for microscopic examination. Formalin-fixed tissues were processed, paraffin-embedded, sectioned at 5 μm, and stained with haematoxylin, phloxine and safranine (HPS) according to standard techniques. Sections were examined by light microscopy for the presence of adhering bacteria on intestinal cells, as previously described (Girard *et al.*, 2005).

## RESULTS AND DISCUSSION

### Expression of isogenic intimin types in CR

Intimin (encoded by *eae*_CR_) is essential both for colonization of mice by CR and for the production of transmissible colonic hyperplasia (TMCH) (Schauer & Falkow, 1993). Strain DBS255 (Δ*eae*_CR_) is completely avirulent; although this phenotype could not be complemented *in trans*, putting the wild-type *eae*_CR_ gene back into the chromosome restored virulence (Schauer & Falkow, 1993). Subsequently a pACYC-borne EPEC *eae_α_* gene (pCVD438) has been shown to complement strain DBS255, restoring virulence and hyperplasia to infected mice (Frankel *et al.*, 1996b). More recently, the *eae* region within pCVD438 encoding Int280*α* was replaced with that of EHEC O157 : H7 *eae_γ_*, producing plasmid pICC55 (Hartland *et al.*, 2000). Significantly, strain DBS255(pICC55) was unable to cause hyperplasia in mice (Hartland *et al.*, 2000).

The aim of this study was to perform direct comparisons between the functionalities of Int280*α*, Int280*β* and Int280*γ* *in vivo*. To this end we replaced the region within pCVD438 encoding Int280*α* with that of CR encoding Int280*β*, producing plasmid pICC327 (Fig. 1). Although some differences in total intimin expression were noted in Western blots (data not shown), the three plasmids (pCDV438, pICC55 and pICC327) are isogenic in that intimin expression is driven from the same, natural, *eae* promoter and the three Int280 domains are presented on the surface from the same intimin platform.

### Effect of intimin type on colonization of C3H/Hej mice – host specificity

**Fig. 2. f2:**
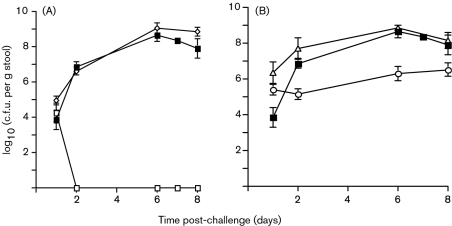
Colonization of mouse gastrointestinal tracts by different CR strains, as indicated by shedding of c.f.u. in stools. The levels of colonization are indicated by the viable bacterial counts (c.f.u., means±sem) from stool samples taken at different times for the 8 days post-challenge. (A) Strain DBS255(pICC327) (◊) was shed in stools at levels very similar to those of the wt strain (▪), whereas strain DBS255 (□) was not recovered after 2 days post-challenge. (B) Strain DBS255(pCVD438) (▵) was shed in stools at a slightly higher level than the wt strain (▪) on days 1 and 2 post-challenge. In contrast, strain DBS255(pICC55) (○) was shed at levels approximately 2 logs lower than those of the wt strain from days 2 to 8 post-challenge.

Mice were challenged orally with 1×10^8^ c.f.u. of the wild-type (wt) strain, the Δ*eae*_CR_ strain DBS255 and DBS255 containing pCVD438 (*eae*280*_α_*), pICC55 (*eae*280*_γ_*) or pICC327 (*eae*280*_β_*). Stool samples were collected during the course of the infection and the numbers of c.f.u. per g stool were determined by plating. The wt strain had a growth curve typical of CR infection of C3H/Hej mice; the number of c.f.u. per g stool slowly increased over the first few days post-inoculation (p.i.), peaking at days 6–8. In contrast strain DBS255 was shed in stools only for the first 24 h p.i. (Fig. 2A[Fig f2]). Plasmid pICC327 (*eae*280*_β_*) fully complemented strain DBS255, restoring colonization and resulting in wt levels of c.f.u. shed in stools over the 8 day infection (Fig. 2A[Fig f2]). Interestingly, plasmid pCVD438 not only fully complemented strain DBS255 in terms of c.f.u. shed in stools, but it colonized mice more efficiently than the wt strain during the first 48 h of infection (Fig. 2B[Fig f2]). This is a reproducible result that we have observed on each of the more than 10 occasions that we have tested this strain in mice. In comparison, strain DBS255(pICC55) had an intermediate colonization phenotype, with 1–2 logs fewer c.f.u. shed in stools over the whole 8 day infection (Fig. 2B[Fig f2]).

**Fig. 3. f3:**
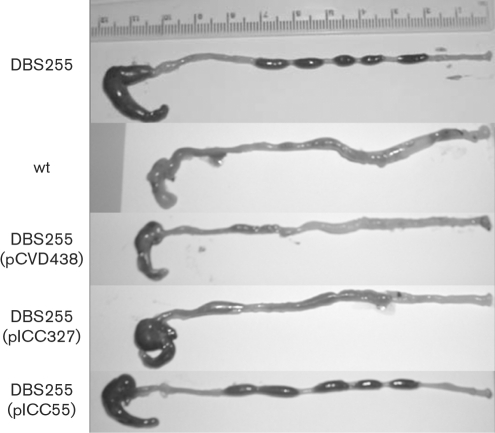
Caeca and colons of mice infected with different CR strains for 8 days. Mice infected with strains DBS255 and DBS255(pICC55) showed a full caecum and well-formed dark stools in the distal colon. In contrast, mice infected with wt, DBS255(pCVD438) and DBS255(pICC327) strains had shorter, thicker colons with a few diffuse watery stools and visible hyperplasia at the distal end. In addition, the caeca in these mice were often half-full or completely empty.

**Fig. 4. f4:**
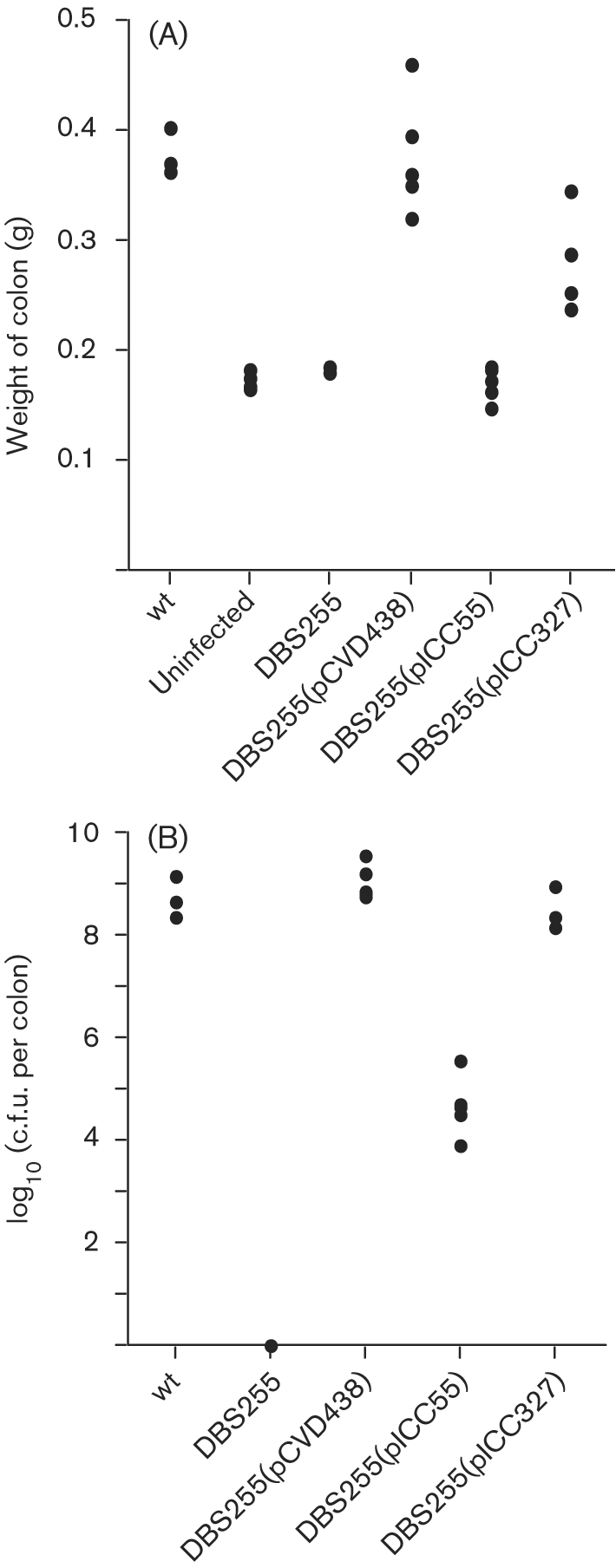
Virulence of CR strains in the mouse colon. (A) The total colon was weighed after the removal of all stools at day 8 post-challenge. Mice infected with DBS255(pCVD438) had colon weights that were not significantly different from those of mice infected with the wt CR. There was no significant difference between the colon weights of mice infected with DBS255 and DBS255(pICC55) and the colon weights of uninfected mice. In contrast, the colon weights of mice infected with strain DBS255(pICC327) were significantly greater than those of uninfected mice (*P*<0.001) but still slightly less than those of mice infected with the wt (*P*<0.05). (b) Mice infected with the wt strain and with DBS255(pCVD438) and DBS255(pICC327) all had similarly high pathogen burdens (around 10^8^–10^9^ c.f.u. per colon). In contrast, mice infected with DBS255(pICC55) had significantly lower bacterial loads (around 10^4^–10^5^ c.f.u. per colon; *P*<0.001), although the levels were still higher than those in mice infected with the DBS255 mutant, from which no challenge bacteria were recovered.

The mice were euthanased at day 8 p.i. and colons were removed for post mortem examination. This time point was chosen as a number of the infected mice had lost 10–15 % of their original body weight and had become almost immobile. The macroscopic appearance of the colons is shown in Fig. 3. Mice infected with DBS255 and DBS255(pICC55) had colons of normal appearance with plenty of dark, well-formed stools, no obvious mucosal thickening and a full caecum. Mice infected with the wt and with strains DBS255(pCVD438) and DBS255(pICC327) all showed visible thickening of the distal colon and only a few pale, diffuse stools. In addition, the caecum was often half-full or entirely empty. The distal 8 cm of colon was washed of stools and weighed to give an indication of degree of hyperplasia (Fig. 4A[Fig f4]). Mice infected with DBS255 and DBS255(pICC55) had colon weights indistinguishable from those of uninfected mice (<0.2 g), whereas wt-infected mice and those infected with DBS255(pCVD438) had colons which were nearly double the weight (0.4 g). Colons from mice infected with DBS255(pICC327) had a mean weight of around 0.3 g, intermediate between wt-infected mice and those infected with DBS255.

The levels of c.f.u. recovered from the colons agreed with those shed in stools, with similarly high levels found in mice infected with wt, DBS255(pCVD438) and DBS255(pICC327); mice infected with DBS255(pICC55) had ∼10^4^ fewer CR bacteria associated with the washed mucosa. No bacteria were recovered from DBS255-infected mice (Fig. 4B[Fig f4]).

### Colonization, protein translocation and A/E lesion formation

**Fig. 5. f5:**
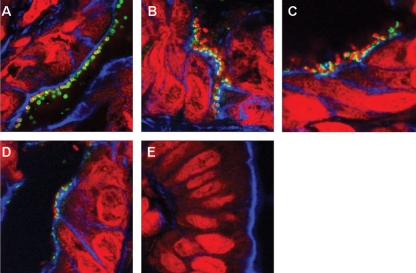
Immunofluorescence staining of CR-infected cryosectioned mouse colons. Staining was performed for CR (A, green) and Tir (B–E, green). Filamentous actin was visualized by phalloidin staining (blue), and bacteria and cell nuclei were counterstained with propidium iodide (red). Intimately adhering bacteria with translocated Tir underneath were observed on mouse colons infected with wt CR (B), DBS255(pICC438) (C) and DBS255(pICC327) (D). No adherent bacteria were detected on mouse colons infected with DBS255(pICC55) (E).

In order to visualize adherent CR bacteria, protein translocation and A/E lesion formation, colonic tissues from infected animals were cryosectioned and processed for immunofluorescence microscopy. Adherent bacteria were confirmed as CR using rabbit CR polyclonal antiserum (Fig. 5A[Fig f5]). Tir translocation and A/E lesions were apparent in tissue taken from mice infected with wt CR, DBS255(pICC438) expressing *eae*280*_α_* and DBS255(pICC327) expressing *eae*280*_β_* (Fig. 5B–D[Fig f5]). In contrast, we could not detect adherent DBS255(pICC55) expressing *eae*280*_γ_* (Fig. 5E[Fig f5]).

These results show that in contrast to a previous report (Schauer & Falkow, 1993) a CR *eae*280*_β_* plasmid can complement CRΔ*eae*, restoring colonization and hyperplasia. CR expressing *eae*280*_α_* is more virulent than the wt CR, with higher levels of colonization in the first few days of infection. In contrast, despite being present in stools at a relatively high number, CR expressing *eae*280*_γ_* did not establish intimate contact with the epithelium and was unable to induce hyperplasia. Considering that all the CR strains are isogenic, that all possess identical type III secretion systems and EspA filaments, and that Int280*γ* binds Tir_CR_ (Hartland *et al.*, 2000), the attenuated phenotype is likely to reflect the absence of a host-cell-encoded intimin *γ* receptor.

In a previous study we showed that CR(pICC55) does not induce hyperplasia (Hartland *et al.*, 2000). However, as colonization was only studied in infected tissue at 12 days p.i., we did not record bacterial shedding at earlier time points. Nevertheless, the current study supports our original conclusion that intimin *γ* is not functionally equivalent to intimin *α* or *β* in the CR model.

### Effect of Int280*β* on tissue specificity – human IVOC

**Fig. 6. f6:**
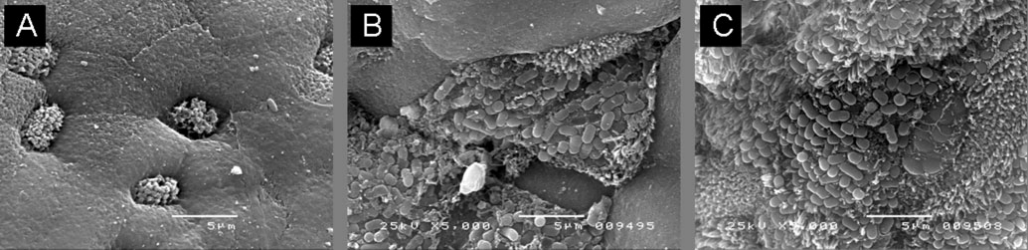
SEM of human IVOC. (A) Non-infected duodenal tissue showed smooth surface epithelium without any bacteria. In contrast, intimately attaching bacteria were present on duodenal mucosa infected with ICC170(pICC327) (B) and CVD206(pICC327) (C). Bars, 5 μm.

Previous studies have shown that exchanging intimins between EPEC O127 : H6 and EHEC O157 : H7 resulted in restriction of EPEC colonization to the Peyer's patch mucosa of human IVOC and extension of colonization of EHEC to proximal small intestine (Fitzhenry *et al.*, 2002a; Phillips & Frankel, 2000). In this study pICC327 was transformed into EPECΔ*eae* (strain CVD206) and EHECΔ*eae* (strain ICC170) mutants. Both CVD206(pICC327) and ICC170(pICC327) adhered to small intestine on 4/4 and 3/4 occasions, respectively (Fig. 6). No adhesion was seen in the *eae*-negative controls (0/4) (data not shown), while the positive controls EPEC E2348/69 and CVD206(pCVD438) adhered to small intestinal mucosa 4/4 and 3/4 times, respectively (data not shown). These results show that like intimin *α*, intimin *β* can also allow colonization of proximal small intestine by EHEC O157 : H7 while EPEC expressing intimin *α* or intimin *β* show similar tissue specificity.

### Effect of Int280*β* on tissue specificity – porcine IVOC

A previous study showed that exchanging intimin *α* and *γ* between EPEC O127 : H6 and EHEC O157 : H7 resulted in restriction of EPEC colonization to the ileal mucosa of porcine IVOC and extension of colonization of EHEC to small intestine (Girard *et al.*, 2005).

**Fig. 7. f7:**
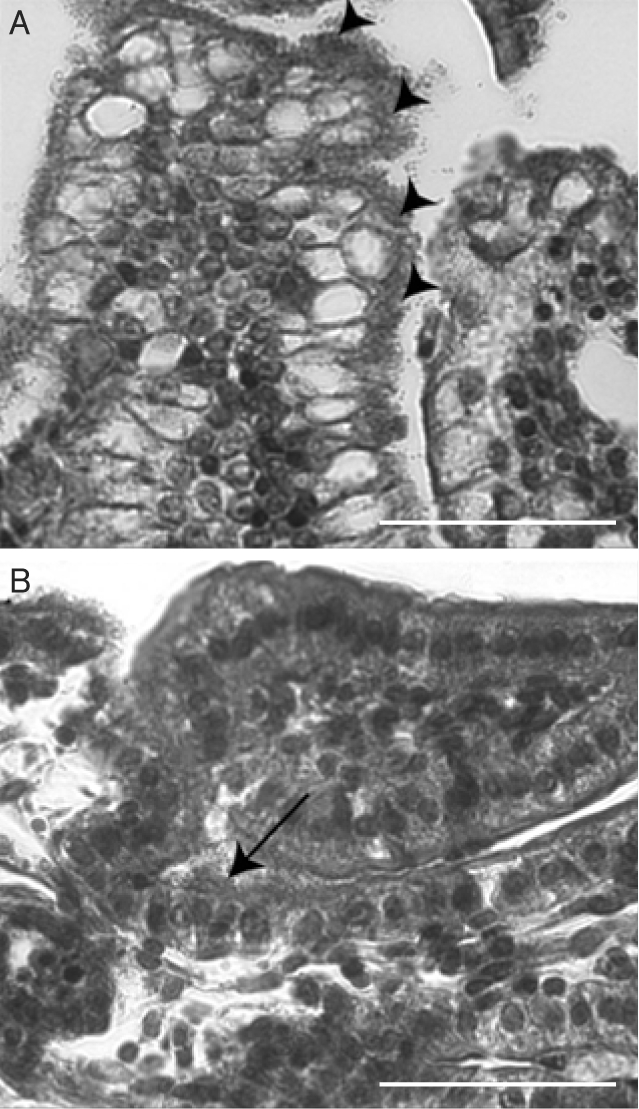
Representative micrographs of HPS-stained sections of porcine IVOC segments inoculated with the complemented mutant strains CVD206(pICC327) (A, ileal IVOC,), or ICC170(pICC327) (B, jejunal IVOC). Large foci of intimately adhering bacteria, along with mucosal irregularities, were observed for CVD206(pICC327) (arrowheads), whereas either smaller foci of intimately adhering bacteria, or individually adhering bacteria, not always associated with mucosal irregularities, were observed for ICC170(pICC327) (arrow). Bars, 500 μm.

**Fig. 8. f8:**
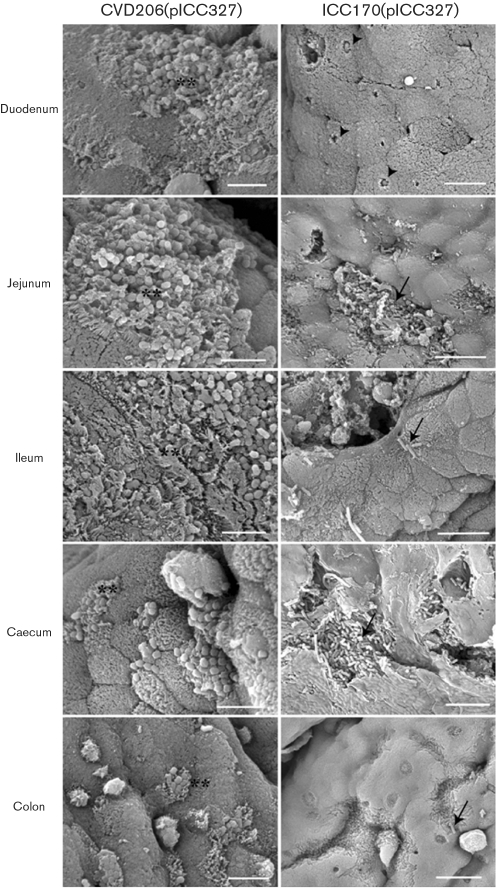
Adherence of the complemented mutant strains CVD206(pICC327) and ICC170(pICC327) to porcine intestinal IVOC prepared from duodenum, jejunum, ileum, caecum and colon, as observed by SEM. Although CVD206(pICC327) induced typical A/E lesions in all intestinal sites investigated (**), complemented mutant strain ICC170(pICC327) showed only rare A/E lesions in the duodenum (arrowheads). Bacteria associated with the epithelium were observed in small to large foci (arrows) in all other intestinal sites investigated, without direct evidence of A/E lesions. Bars, 5 μm [CVD206(pICC326) and ICC170(pICC327) duodenum] or 10 μm [ICC170(pICC327) jejunum, ileum, caecum and colon].

In order to determine the functionality of intimin *β* during infection of porcine IVOC, biopsies taken from different sites were infected with recombinant EPECΔ*eae* and EHECΔ*eae* strains. Observation of HPS-stained sections showed that CVD206(pICC327) adhered to all parts of the small intestine at a comparable level to that seen with E2348/69 (Table 2), whereas only a few sites with adhering bacteria were observed in the caecum and the colon (Table 2). For its part, adherence of ICC170(pICC327) was mostly observed in the jejunum, whereas few sites with adhering bacteria were observed in the ileum, caecum and colon (Table 2). Foci of small to large aggregates of adherent bacteria were observed for CVD206(pICC327) (Fig. 7A[Fig f7]), whereas relatively small foci or individual adherent bacteria were observed on epithelial cells for ICC170(pICC327) (Fig. 7B[Fig f7]). Loose association of bacteria with the intestinal mucosa of some villi, with no obvious change in associated epithelial cell morphology, was observed for the *eae* mutants CVD206 and ICC170 (data not shown), as previously described (Girard *et al.*, 2005). SEM analysis of the mucosal surface of whole explants inoculated with CVD206(pICC327) demonstrated typical A/E lesions and gross microvillous elongation in the duodenum, jejunum and ileum, whereas the caecum and colon were more slightly colonized, but still demonstrated some A/E lesions (Fig. 8). On the other hand, explants inoculated with ICC170(pICC327) demonstrated only rare A/E lesions with very localized effacement and no microvillous elongation in the duodenum and jejunum (Fig. 8); in all other intestinal segments examined the bacteria were associated with the epithelial cells in small or large aggregates, with no direct evidence of A/E lesions (Fig. 8).

These results show that while intimin *β* in EPECΔ*eae* can completely restore colonization of porcine IVOC, the expression of intimin *β* in EHECΔ*eae* does not restore colonization potential fully. This is unlikely to be due to incompatibility between intimin_CR_ and Tir_EHEC_, as EHEC expressing Int_CR_ was functional during infection of human IVOC (Fig. 6), and Deng *et al*. (2003) have shown that Tir_CR_ and Tir_EHEC_ are interchangeable.

These results show, as we have shown before for EPEC O55 (Fitzhenry *et al.*, 2002b), that determination of host and tissue specificity by A/E-lesion-forming *E. coli* is multifactorial, involving other bacterial and host determinants, as well as intimin.

## Figures and Tables

**Table 1. t1:** Plasmids used in this study

**Plasmid**	**Description**	**Reference**
pACYC184	Cm^R^ Tc^R^ medium-copy-number cloning vector	New England Biolabs
pCVD438	pACYC184 encoding EPEC intimin *α*	Donnenberg & Kaper (1991)
pICC55	pCVD438 derivative encoding recombinant intimin *γ*	Hartland *et al*. (2000)
pICC327	pCVD438 derivative encoding recombinant intimin *β*	This study

**Table 2. t2:** Adherence of EPEC and EHEC strains to porcine intestinal explants

**Strain**	**Duodenum**	**Jejunum**	**Ileum**	**Caecum**	**Colon**
E2348/69	13/13 (100 %)	13/13 (100 %)	11/11 (100 %)	6/6 (100 %)	1/6 (16.7 %)
CVD206	1/11* (9.1 %)	5/10 (50 %)	3/11 (27.3 %)	0/11 (0 %)	0/6 (0 %)
CVD206(pICC327)	10/12 (83.3 %)	12/12 (100 %)	12/12 (100 %)	4/12 (33.3 %)	1/12 (8.3 %)
85-170	0/6 (0 %)	2/6 (33.3 %)	14/15 (93.3 %)	3/6 (50 %)	0/6 (0 %)
ICC170	0/6 (0 %)	0/6 (0)	14/18* (77.8 %)	0/6 (0 %)	0/6 (0)
ICC170(pICC327)	0/11 (0 %)	5/11 (45.5 %)	3/12 (25.0 %)	4/12 (33.3 %)	3/12 (25.0 %)

*Although adhering bacteria were observed on HPS-stained sections, SEM demonstrated no A/E lesions for both Δ*eae* strains CVD206 and ICC170 in all intestinal sites assessed.

## Abbreviations

- A/E, attaching and effacing
- CR, *Citrobacter rodentium*
- EHEC, enterohaemorrhagic *E. coli*
- EPEC, enteropathogenic *E. coli*
- IVOC, *in vitro* organ culture
- HPS, haematoxylin, phloxine and safranine
- LEE, locus of enterocyte effacement
- p.i., post-inoculation
- SEM, scanning electron microscope/microscopy
